# Supplementary material for: Unlocking Cellular Memory and Gene Regulatory Networks: Pioneering the Future of Therapeutic Innovations
Source: Cells. 2025 Jun 14;14(12):903. doi: 10.3390/cells14120903 (PMC12191380; doi:10.3390/cells14120903)
Supplement: Supplementary file 1 [file cells-14-00903-s001.zip › cells-3643000-supplementary.pdf]

Supplementary Information

Unlocking Cellular Memory and Gene Regulatory Networks: Pioneering the Future of Therapeutic Innovations

Md Sorique Aziz Momin[1,†], **Jhuma Bhadra**[2,†], **Debmalya Bhunia**[3,†], **Achinta Sannigrahi**[4,*], **Nayan De**[5,*]

[1]School of Physics and Astronomy, College of Science, Rochester Institute of Technology, Rochester, NY 14623, USA
[2]Department of Chemistry, Sarojini Naidu College for Women, Kolkata 700028, India
[3]Cold Spring Harbor Laboratory, 1 Bungtown Rd, Cold Spring Harbor, NY 11724, USA
[4]University of Texas Southwestern Medical Center, 5323 Harry Hines Blvd, Dallas, TX 75390, USA
[5]Institute for System Biology, 401 Terry Ave N, Seattle, WA 98109, USA

†Equal contribution
*Corresponding authors.
E-mail: sannigrahiachinta@gmail.com (Dr. Sannigrahi)
E-mail: nde@systemsbiology.org, nayanchemju1@gmail.com (Dr. De)

1 Kinetic Model of Gene Transcription Regulation

A Gene Transcription Regulatory Network (GTRN) motif can be described in terms of the concentration or copy number of its constituent gene products. For instance, consider a gene product X synthesized at a constant rate k_x and degraded with a first-order rate constant μ_x. These processes contribute to changes in the gene expression profile over time. In standard chemical kinetics notation, these processes are represented as follows [1]:

$$\emptyset \overset{k_x}{\to} X \overset{\mu_x}{\to} \emptyset.$$

Here, the arrows indicate chemical reactions, not transcriptional activation. The ordinary differential equation that describes the deterministic dynamics of the copy number of X is given by:

$$\frac{dX}{dt} = k_x - \mu_x X. \tag{S1}$$

At steady state, the production and degradation of X are balanced, and the maximum copy number reaches $X(t \to \infty) = \frac{k_x}{\mu_x}$.

When transcriptional factors (TFs) regulate the synthesis of gene products, the model can be described using mass-action kinetics similar to enzyme-substrate reactions. In the case of activation, a TF X enhances the rate of production of a protein Y when it binds to the promoter region of Y's DNA (D) to form a complex $[XD]$. From this complex, protein Y is produced. Given the close coupling of transcription and translation processes, we model these as a single reaction step due to the significant timescale difference between transcription ($\sim$ minutes) and translation ($\sim$ hours). The kinetics of protein Y production and degradation are described as [1]:

$$\underset{\text{Activator TF}}{X} + \underset{\text{DNA}}{D} \underset{k_{\text{off}}}{\overset{k_{\text{on}}}{\rightleftharpoons}} \underset{\text{TF-DNA complex}}{[XD]} \overset{k_{xy}}{\to} X + D + \underset{\text{Protein}}{Y},$$
$$Y \overset{\mu_y}{\to} \emptyset.$$

Here, k_{on} and k_{off} represent the binding and unbinding rates of X to its target promoter, respectively, while k_{xy} and μ_y denote the production and degradation rates of protein Y. The dynamics of the TF-DNA complex

and the protein Y are governed by the following equations:

$$\frac{d[XD]}{dt} = k_{\text{on}} XD - (k_{\text{off}} + k_{xy})[XD], \tag{S2}$$

$$\frac{dY}{dt} = k_{xy}[XD] - \mu_y Y. \tag{S3}$$

Since the binding and unbinding of X to its target promoter occurs much faster than the protein production step ($\{k_{\text{on}}, k_{\text{off}}\} \gg k_{xy}$), the TF-DNA complex can be considered to reach a quasi-steady state on the timescale of Y production. Thus, the fraction of DNA bound to X at steady state is given by [2, 1]:

$$\frac{[XD]}{D_T} = [XD] = \frac{k_{\text{on}} X}{k_{\text{off}} + k_{xy} + k_{\text{on}} X} \approx \frac{X}{(k_{\text{off}}/k_{\text{on}}) + X} = \frac{X}{K_{xy} + X}. \tag{S4}$$

In this equation, $K_{xy} = k_{\text{off}}/k_{\text{on}}$ represents the dissociation or Michaelis-Menten constant, analogous to the activation/repression coefficient in gene regulation models. The binding affinity of the regulatory protein to its target promoter is inversely related to K_{xy}. By substituting Eq. 4 into Eq. 3, we obtain:

$$\frac{dY}{dt} = k_{xy} \frac{X}{K_{xy} + X} - \mu_y Y. \tag{S5}$$

The ratio on the right-hand side of this equation is known as the Hill function, which quantifies the occupancy probability of the promoter Y by the regulator X. The term K_{xy} represents the copy number of X required to achieve half-maximal gene expression of Y. When $K_{xy} = X$, transcriptional control is effectively established. The domains $K_{xy} \gg X$ and $K_{xy} \ll X$ correspond to first-order and zero-order activation kinetics, respectively. The production rate parameter k_{xy} denotes the maximum promoter activity. At high activator concentrations, Y production increases due to enhanced activity of RNA polymerase in transcribing the gene. Activation can also be enhanced by a decreased value of K_{xy}.

In the case of repression, the TF inhibits transcription by binding to the target promoter and forming a complex $[XD]$, thereby blocking RNA polymerase from attaching to the DNA. The repression kinetics are given by [2, 1]:

$$\underset{\text{Repressor TF}}{X} + \underset{\text{DNA}}{D} \underset{k_{\text{off}}}{\overset{k_{\text{on}}}{\rightleftharpoons}} \underset{\text{TF-DNA complex}}{[XD]},$$

$$D \overset{k_{xy}}{\rightarrow} D + \underset{\text{Protein}}{Y}.$$

Assuming a quasi-steady state as before, we obtain:

$$\frac{dY}{dt} = k_{xy} \frac{K_{xy}}{K_{xy} + X} - \mu_y Y. \tag{S6}$$

When multiple identical TFs form multimers to regulate their target gene, the transcriptional dynamics are described by:

$$\frac{dY}{dt} = k_{xy} \frac{X^n}{K_{xy}^n + X^n} - \mu_y Y \text{ (activation)}, \tag{S7}$$

$$\frac{dY}{dt} = k_{xy} \frac{K_{xy}^n}{K_{xy}^n + X^n} - \mu_y Y \text{ (repression)}. \tag{S8}$$

Here, n represents the cooperativity index or Hill coefficient, which influences the steepness of the production term. The typical range for n spans from 1 (hyperbolic control) to 30 (switch-like behavior). For simplicity, we do not consider cooperative effects in the Hill functions. In practical scenarios, there is often a small basal level of gene product production even in the absence of transcriptional activation or repression. However, since this basal production is much smaller than the regulatory effects, it is not included in our models. Beyond the Hill function-based kinetic models, alternative approaches incorporating thermodynamic regulation factors from equilibrium statistical mechanics are available. In our case, Eqs. (1, 5) and Eqs. (1, 6) represent the deterministic activation and repression dynamics of Y by X, respectively, denoted symbolically by $X \rightarrow Y$ and $X \dashv Y$.

1.1 Positive autoregulation loop

Positive autoregulation occurs when a protein, S, enhances its own production by activating its own transcription. In this feedback loop, S binds to its gene's promoter, increasing its own synthesis. The dynamics are described by [3, 4, 5]:

$$\frac{dS}{dt} = k_{ss}\frac{S^n}{K_{ss}^n + S^n} - \mu_s S + \xi_s(t) \tag{S9}$$

Here, k_{ss} is the maximum production rate of S, K_{ss} is the half-maximal concentration for activation, n is the Hill coefficient, and μ_s is the degradation rate. This mechanism creates a self-reinforcing cycle that can lead to bistability, where S can be in a high or low concentration state depending on initial conditions.

1.2 Double positive feedback loop

In a double positive feedback loop, the dynamics of two interacting components, X and Y, are described by equations that capture the mutual enhancement of each other's production. The production rate of X is positively influenced by Y, while X undergoes degradation or dilution. Similarly, Y is produced in response to X and also faces degradation. The equations governing these dynamics reflect the Hill function, which quantifies the effect of one component on the production of the other. For X, the production rate due to Y is represented by a term that increases as Y rises, modulated by a Hill coefficient and a half-maximal concentration parameter, with degradation reducing the X concentration. Conversely, the dynamics for Y follow a similar pattern, driven by X and influenced by its own degradation rate. The resulting equations are as follows [3, 4, 5]:

$$\frac{dX}{dt} = k_{xy}\frac{Y^n}{K_{xy}^n + Y^n} - \mu_x X + \xi_x(t) \tag{S10}$$

$$\frac{dY}{dt} = k_{yx}\frac{X^n}{K_{yx}^n + X^n} - \mu_y Y + \xi_y(t) \tag{S11}$$

In these equations, k_{xy} and k_{yx} are the maximum production rates of X and Y due to the presence of Y and X, respectively. K_{xy} and K_{yx} are the half-maximal concentrations for these activations, n is the Hill coefficient indicating cooperativity, and μ_x and μ_y are the degradation rates of X and Y.

2 Analytical expressions for (Co)variances

The noise terms $\xi_x(t)$ and $\xi_y(t)$ are modeled as independent white Gaussian noise, characterized by $\langle \xi_i(t)\rangle = 0$ and $\langle \xi_i(t)\xi_j(t')\rangle = \langle |\xi_i|^2\rangle \delta_{ij}\delta(t-t')$, where $\langle \cdots \rangle$ denotes the steady-state ensemble average [6, 7, 8, 9, 10]. The noise strengths, which arise equally from the synthesis and degradation of the biochemical species at steady state, are given for species X as $\langle |\xi_x|^2\rangle = \langle f_x\rangle + \mu_x\langle X\rangle = 2\langle f_x\rangle$. This is an approximation, implying that $\langle |\xi_x|^2\rangle = 2\mu_x\langle X\rangle$ [11, 10, 12, 13]. A similar approach is used to model the steady-state noise strength for Y.

We linearize the set of Eqs. (10-11) at steady state by considering small perturbations of the form $\delta Z(t) = Z(t) - \langle Z\rangle \ll \langle Z\rangle$ for $Z \in \{X, Y\}$ [14, 9, 8, 15]. We use the Lyapunov equation to connect fluctuations and dissipation in the system and extract the variances and covariances of the gene products as follows [16, 17]:

$$\boldsymbol{J\Sigma} + \boldsymbol{\Sigma J}^T + \boldsymbol{D} = \boldsymbol{0}, \tag{S12}$$

with

$$
\begin{aligned}
\boldsymbol{J} &= \begin{pmatrix} \langle f'_{x,y}\rangle & -\mu_x \\ \langle f'_{y,x}\rangle & -\mu_x \end{pmatrix}, \\
\boldsymbol{\Sigma} &= \begin{pmatrix} \Sigma(X) & \Sigma(X,Y) \\ \Sigma(Y,X) & \Sigma(Y) \end{pmatrix}, \\
\mathbf{D} &= \begin{pmatrix} \langle|\xi_x|^2\rangle & 0 \\ 0 & \langle|\xi_y|^2\rangle \end{pmatrix}.
\end{aligned}
$$

Here, $\boldsymbol{J}$ and $\boldsymbol{\Sigma}$ are the Jacobian and (co)variance matrices, respectively. The term $\langle f'_{x,y}\rangle$ denotes $\left(\frac{df_x(Y)}{dY}\right)_{Y=\langle Y\rangle}$, where $f_x(Y) = k_{xy}\frac{Y^n}{K_{xy}^n+Y^n}$, and similarly for other terms. Given that $\Sigma(X,Y) = \Sigma(Y,X)$ and similar relations, $\boldsymbol{\Sigma}$ is a symmetric matrix. The symbol T denotes matrix transposition. The matrix $\boldsymbol{D}$ contains various steady-state noise strengths and is diagonal due to the lack of noise correlation between different biochemical species. Solving Eq. (12) for $\boldsymbol{\Sigma}$ yields the required expressions.

$$\Sigma(X) = -\frac{f'_{xy}f'_{yx}\langle X\rangle\mu_x - (f'_{xy})^2\langle Y\rangle\mu_y - \langle X\rangle\mu_x^2\mu_y - \langle X\rangle\mu_x\mu_y^2}{(\mu_x+\mu_y)(\mu_x\mu_y - f'_{xy}f'_{yx})} \tag{S13}$$

$$\Sigma(X,Y) = \frac{\mu_x(f'_{yx}\langle X\rangle\mu_y + f'_{xy}\langle Y\rangle\mu_y)}{(\mu_x+\mu_y)(\mu_x\mu_y - f'_{xy}f'_{yx})} \tag{S14}$$

$$\Sigma(Y) = \frac{(f'_{yx})^2\langle X\rangle\mu_x - f'_{xy}f'_{yx}\langle Y\rangle\mu_y + \langle Y\rangle\mu_x^2\mu_y + \langle Y\rangle\mu_x\mu_y^2}{(\mu_x+\mu_y)(\mu_x\mu_y - f'_{xy}f'_{yx})} \tag{S15}$$

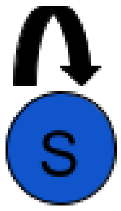

(a)

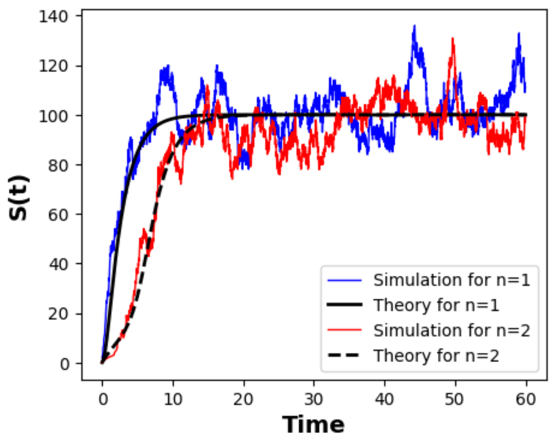

(b)

Figure 1: Numerical simulation of gene expression S regulated by self-produced transcription factors [18, 19]. The blue line represents the case with a Hill coefficient $n = 1$, while the red line corresponds to $n = 2$. Black lines indicate analytical solutions. The parameters used in the simulation are $k_{ss} = 5\,\mathrm{min}^{-1}$, $\mu_s = 0.5\,\mathrm{min}^{-1}$, and $K_{ss} = \langle S\rangle$ (copy numbers).

A positive autoregulation loop, where a gene, say S, enhances its own expression, creates a self-reinforcing cycle that amplifies and stabilizes its production. For a synthesis rate constant k_{ss} and a degradation rate parameter μ_s, the gene expression level of S reaches 100 copy numbers when the cooperativity index n is set at 1 and 2, as shown in Figure 1. We observe that the gene expression level for $n = 1$ reaches a steady state faster than the gene expression for $n = 2$.

It has been found in earlier research that positive autoregulation has the opposite effect of negative autoregulation: it slows the response time relative to simple regulation. The dynamics are initially slow, but

as the levels of S build up, S increases its own production, reaching halfway to steady state with a delay compared to simple regulation. In our research, we observe that within a positive autoregulation loop, the response time slows down even further for $n = 2$ compared to $n = 1$.

Positive autoregulation not only provides slow dynamics, which are beneficial in multi-stage processes requiring extended durations, such as developmental processes, but it also introduces delays between the production of proteins that drive different stages. Additionally, positive autoregulation has a remarkable ability to make sharp decisions between two distinct states and retain that decision for an extended period, adding a memory-like feature to the system.

References

[1] Uri Alon. *An Introduction to Systems Biology: Design Principles of Biological Circuits, 2nd ed.* CRC press, 2020.

[2] L. Bintu et al. "Transcriptional regulation by the numbers: models". In: *Curr. Opin. Genet. Dev.* 15 (2005), pp. 116–124.

[3] M. S. A. Momin, A. Biswas, and S. K. Banik. "Coherent feed-forward loop acts as an efficient information transmitting motif". In: *Phys. Rev. E* 101 (2020), p. 022407.

[4] M. S. A. Momin and A. Biswas. "Extrinsic noise of the target gene governs abundance pattern of feed-forward loop motifs". In: *Phys. Rev. E* 101 (2020), p. 052411.

[5] Md Sorique Aziz Momin and Ayan Biswas. "The role of gene regulation in redundant and synergistic information transfers in coherent feed-forward loop". In: *Journal of Statistical Mechanics: Theory and Experiment* 2023.2 (Feb. 2023), p. 023501. ISSN: 1742-5468. DOI: 10.1088/1742-5468/acb42e. URL: http://dx.doi.org/10.1088/1742-5468/acb42e.

[6] S. Tănase-Nicola, P. B. Warren, and P. R. ten Wolde. "Signal detection, modularity, and the correlation between extrinsic and intrinsic noise in biochemical networks". In: *Phys. Rev. Lett.* 97 (2006), p. 068102.

[7] P. B. Warren, S. Tănase-Nicola, and P. R. ten Wolde. "Exact results for noise power spectra in linear biochemical reaction networks". In: *J. Chem. Phys.* 125 (2006), p. 144904.

[8] N G van Kampen. *Stochastic Processes in Physics and Chemistry, 3rd ed.* North-Holland, Amsterdam, 2007.

[9] J. Elf and M. Ehrenberg. "Fast evaluation of fluctuations in biochemical networks with the linear noise approximation". In: *Genome Res.* 13 (2003), pp. 2475–2484.

[10] A. Biswas and S. K. Banik. "Redundancy in information transmission in a two-step cascade". In: *Phys. Rev. E* 93 (2016), p. 052422.

[11] W. H. de Ronde, F. Tostevin, and P. R. ten Wolde. "Feed-forward loops and diamond motifs lead to tunable transmission of information in the frequency domain". In: *Phys. Rev. E* 86 (2012), p. 021913.

[12] A. Biswas and S. K. Banik. "Interplay of synergy and redundancy in diamond motif". In: *Chaos* 28 (2018), p. 103102.

[13] A. Biswas. "Multivariate information processing characterizes fitness of a cascaded gene-transcription machinery". In: *Chaos* 29 (2019), p. 063108.

[14] J Keizer. *Statistical Thermodynamics of Nonequilibrium Processes.* Springer-Verlag, Berlin, 1987.

[15] R Grima. "Linear-noise approximation and the chemical master equation agree up to second-order moments for a class of chemical systems". In: *Phys. Rev. E* 92 (2015), p. 042124.

[16] J Paulsson. "Summing up the noise in gene networks". In: *Nature* 427 (2004), pp. 415–418.

[17] J Paulsson. "Models of stochastic gene expression". In: *Phys Life Rev* 2 (2005), pp. 157–175.

[18] D T Gillespie. "A general method for numerically simulating the stochastic time evolution of coupled chemical reactions". In: *J. Comp. Phys.* 22 (1976), pp. 403–434.

[19] D T Gillespie. "Exact stochastic simulation of coupled chemical reactions". In: *J. Phys. Chem.* 81 (1977), pp. 2340–2361.
